# Reinforced L-Shaped Frame Made of Textile-Reinforced Concrete

**DOI:** 10.3390/polym15020376

**Published:** 2023-01-10

**Authors:** Jiří Žalský, Tomáš Vlach, Jakub Řepka, Jakub Hájek, Petr Hájek

**Affiliations:** 1 Klokner Institute, Czech Technical University in Prague, 16608 Praha, Czech Republic; 2 University Centre for Energy Efficient Buildings, Czech Technical University in Prague, 27343 Bustehrad, Czech Republic; 3 Faculty of Civil Engineering, Czech Technical University in Prague, 16629 Praha, Czech Republic

**Keywords:** textile-reinforced concrete, high-performance concrete, carbon fibers, rigid frame, numerical simulations

## Abstract

Textile-reinforced concrete is becoming more and more popular. The material enables the realization of very thin structures and shells, often with organic shapes. However, a problem with this reinforcement occurs when the structure is bent (contains a corner), and the flexural stiffness around this bent area is required. This article presents the design, solution, and load-bearing capacity of an L-shaped rigid frame made of textile-reinforced concrete. Basic material parameters of concrete matrix and carbon textile reinforcement were supplemented by a four-point bending test to calibrate fracture energy Gf, critical compressive displacement Wd, solver type, and other parameters of a numerical model created by Atena Engineering in specialized non-linear structural analysis software for reinforced concrete structures. The calibrated numerical model was used to evaluate different variants of carbon textile reinforcement of the L-shaped frame. The carbon textile reinforcement was homogenized using epoxy resin to ensure the interaction of all fibers, and its surface was modified with fine-grained silica sand to increase the cohesion with the concrete matrix. Specimens were produced based on the most effective variant of the L-shaped frame reinforcement to be experimentally tested. Thanks to the original shaping and anchoring of the reinforcement in the corner area, the frame with composite textile reinforcement is rigid and can transmit the bending stresses in both positive and negative directions. The results of the mechanical loading test on small experimental specimens correspond well to the results of numerical modeling using Atena Engineering software.

## 1. Introduction

Exposed concrete without plaster or any other layers is becoming very popular in architecture and civil engineering [1]. It is commonly used as a load-bearing structure, as well as a non-bearing design for interior and exterior elements. When designing these often very thin and lightweight structures, the main limiting factor is the concrete cover layer required for traditional steel reinforcement protection. Textile-reinforced concrete (TRC) provides an excellent alternative [2,3,4,5,6]. TRC is reinforced by technical textiles made of inorganic fibers such as carbon, alkali-resistant glass, basalt, etc. These materials are not subject to corrosion, and there is no need for a large concrete cover layer as with traditional reinforced concrete. The concrete cover must only ensure sufficient interaction between the textile reinforcement and the cementitious matrix. In textile reinforcement, the fibers are concentrated into strands called rovings, which are processed into technical textiles for non-aesthetic purposes–grids with specific spacing. Grids are placed on the tensioned side of concrete structures similarly to traditional steel reinforcement in one or more layers [7].

This paper is focused on curved structures made of TRC. Particular attention is focused on the corner of the L-shaped frame and the design of its reinforcement without affecting the thickness of the element and its bending stiffness. Realizations with an organic shape are quite common these days [1,5,8,9], but the textile reinforcement is shaped to conform to the geometry of the elements, not to provide sufficient reinforcement for more complex details such as corners. This approach leads to an insufficient anchoring length of the reinforcement on the inside of the corner, and there is a risk of it being pulled out. Some publications on textile concrete focus on corner reinforcement but exclusively as an additional strengthening of existing steel-reinforced structures [10,11]. The main goal of corner reinforcement, in general, is the successful anchoring of the inner tensile reinforcement bars on the opposite outer surface of the corner with compressive stress to ensure stability against corner openings. In this respect, the presented design of textile reinforcement is close to the traditional approach in steel-reinforced structures [12,13,14].

A rigid frame is generally a very complex and advanced part of a structure where vertical and horizontal structures are connected, and internal forces must be transmitted. In a rigid frame, the Bernoulli–Navier hypothesis (B-region) does not apply, and it has to be considered as a discontinuity region (D-region). D-regions can be designed in many ways in the field of concrete structures. The reinforcement can be designed based on experiences, calculated using strut-tie models, by numerical analysis, or verified by experiments [15,16]. In this case, the presented L-shaped rigid frame was calculated by numerical analysis using Atena Engineering software and later verified by experiments.

Accurate material parameters were needed for the numerical analysis of the rigid frame. A number of associated experiments were conducted to obtain the necessary data. The presented experiments include only impregnated carbon textile reinforcement. Carbon rovings were impregnated with epoxy resin to ensure the homogenization of individual fibers [17]. To validate and calibrate the model inputs, a sub-model of a four-point bending test of a thin TRC plate was created in the Atena Engineering software supported by mechanical experiments. The calibrated sub-model was used as a basis for the more complex modeling of the L-shaped rigid frame with textile reinforcement.

The L-shaped rigid frame was designed to accurately represent the scale of applications for which the textile reinforcement was mainly intended, such as stair steps, façade panels, and reinforcing ribs of the shell structures. Several different types of rigid frame reinforcement were proposed. Each textile reinforcement design was evaluated by Atena Engineering software, and load-bearing capacity and crack development were compared. Mechanical loading was performed only on the specimens based on the most suitable design according to the numerical analysis. Finally, the results of the experiments were compared with the results of the numerical analysis, and the characteristic values of the bearing capacity were determined.

## 2. Materials and Methods

### 2.1. Materials Used

#### 2.1.1. Concrete

The HPC mixture used in this experiment was developed at the Faculty of Civil Engineering, Czech Technical University in Prague (FCE CTU) for various applications [18]. This mixture was designed using mainly local sources of raw materials. It is a self-compacting fine-grained concrete, and its composition is presented in Table 1. The HPC mixture used in this experiment was without any types of fibers. The water–cement ratio was 0.25, and the water–binder ratio was 0.20 for this mixture. Compressive strength tested on cubes with sides of 100 mm was equal to 138.2 ± 1.6 MPa according to the standard CSN EN 12390-3. Tensile strength while bending tested on beams with dimensions of 160 × 40 × 40 mm, was equal to 8.54 ± 0.5 MPa according to standard CSN EN 12390-5. The same HPC recipe has also been used for several applications and research activities at the CTU, such as waffle and solid experimental facade elements [19,20]. Using the same concrete mixture allows the results to be compared with each other during the continuous process of alternative reinforcement development.

#### 2.1.2. Reinforcement

Textile reinforcement was produced manually from carbon fiber homogenized with epoxy resin. The rovings used were from the company Tenax^®^ STS40 F13 24K (Teijin, Tokyo, Japan) with a length weight (titer) of 1 600 g/km (=1 600 tex), a tensile strength of 4400 MPa, and a modulus of elasticity of 240 GPa, according to the technical data sheet. Epoxy resin SikaFloor-150^®^ from the company (Sika, Brno, Czech Republic) was used for the homogenization of the rovings. The basic parameters of pure resin are tensile strength in bending of 15 MPa and modulus of elasticity of 2.0 GPa. The specific gravity of the material is 1 100 kg/m^3^, according to the technical data sheet. This resin has excellent penetrating properties due to its low viscosity and is, therefore, very suitable for roving homogenization. The two-component resin hardens at room temperature. Fine-grained silica sand with grain sizes from 0.1 mm to 0.6 mm was used for surface modification to increase cohesion with the concrete matrix.

Homogenization of the carbon rovings was necessary to ensure the utilization of all fibers since the concrete matrix cannot penetrate deep enough between the carbon fibers. Without homogenization, the core of the roving would interact only through friction between the fibers, which would effectively lower the tensile strength of the reinforcement. Homogenization of the carbon rovings was performed in the laboratory using a foam roller for epoxy resin application. This method resulted in higher volumetric content of epoxy resin of around 65% compared to commercially available products because the excess resin was not squeezed out. Fine-gained silica sand was poured over freshly impregnated rovings. Grains of sand stuck to the epoxy resin on the surface of the impregnated carbon rovings.

### 2.2. Numerical Model Calibration

#### 2.2.1. Performed Experiments for Material Parameters

Many material parameters were required for correct numerical analysis, so experiments were performed to determine basic properties such as the compressive and tensile strength of concrete, the tensile strength of carbon roving, and the cohesion between the concrete matrix and the impregnated carbon roving. Experiments were performed at the University Centre for Energy Efficient Buildings of Czech Technical University in Prague (UCEEB CTU). The experiments regarding the carbon textile impregnated with epoxy resin were performed in two variants to evaluate the effects on cohesion. The first variant was with the smooth untreated surface of the impregnated rovings, while the second was with surface treatment using a fine-grained silica sand [17,21].

The compressive strength of concrete was measured using Controls MCC-Multitest testing machine according to CSN EN 12390-3 on three cubes with sides of 100 mm and was equal to 138.2 ± 1.6 MPa. The tensile strength of concrete was measured on three prisms with dimensions of 160 × 40 × 40 mm and was equal to 8.54 ± 0.5 MPa according to CSN EN 12390-5. The tensile strength of carbon reinforcement was tested using LabTest 4.100SP1 testing machine with a constant increment of displacement of 1.2 mm/min on rovings impregnated with epoxy resin. Impregnation of rovings ensured proper interaction of all fibers [17]. The ultimate tensile strength was dependent on the quality of resin impregnation, as improperly coated fibers broke prematurely, and the tensile strength decreased. The theoretical and measured parameters of the materials are presented in Table 2. Seven specimens were tested for each variant.

In addition to the basic properties of the materials, it was necessary to verify their mutual interaction. Experiments set to determine cohesion between impregnated carbon rovings and cementitious matrix were performed. Two sets with different surfaces were prepared—one with a smooth surface and the other with a surface modified with fine-grained silica sand. The impregnated rovings were encased in an HPC board with a thickness of 20 mm, and one of their ends was subjected to load controlled by displacement of 1 mm/min using LabTest 4.100SP1 testing machine (LaborTech Ltd., Opava, Czech Republic). The pull-out of the reinforcement was measured by a potentiometer on the free end of the reinforcement. The most important parameter was the value of the force when the reinforcement started slipping. Based on the results, a diagram was created that could be used to set the bonding properties in the numerical analysis [17]. Due to almost immediate slipping in the case of smooth-surfaced reinforcement, only reinforcement with a modified surface was chosen for further use.

#### 2.2.2. Calibration

Based on the properties of the materials and their interaction, a numerical model was created as a nonlinear analysis of the finite element method (FEM) in the ATENA software [22,23]. The model was designed in a GiD preprocessor, which is used to prepare the geometry and create a finite element mesh. Material parameters and boundary conditions were set in the ATENA module. Fifteen iterations of the numerical model were created to properly calibrate parameters that were not obtained by testing. Material parameters, such as the compressive strength of concrete, the tensile strength of concrete, the tensile strength of roving, the modulus of elasticity of roving, and the bond stiffness were the same for all the models, while fracture energy G_f_, critical compressive displacement W_d_, solver type and other parameters have been changed to achieve the best representation of a four-point bending test which was used to calibrate the model.

**Figure 1:**
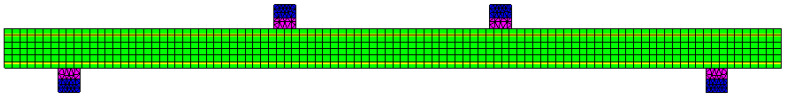
2D basic model of a concrete slab for the four-point bending test created in GiD software. Distance of support is 300 mm; distance of loading support is 100 mm.

The numerical model for calibration was created as a 2D simply supported structure. The effect of symmetry was not considered. The model was the same as the sample dimensions for the experiment of the four-point bending test. The depth of the slab was set as a parameter of the material. The finite element mesh of the structure was made of hexahedra quadratic bricks, with six of them representing the thickness of the structure. Supports for corresponding element activation were modeled with a soft insert based on previous experience because of the more accurate crack development in the model [24]. The reinforcement was modeled as 1D polylines with assigned interaction conditions and its diameter, and other parameters were set as material properties. The model was loaded by constant displacement, similar to the four-point bending test. The model is presented in Figure 1.

**Figure 2:**
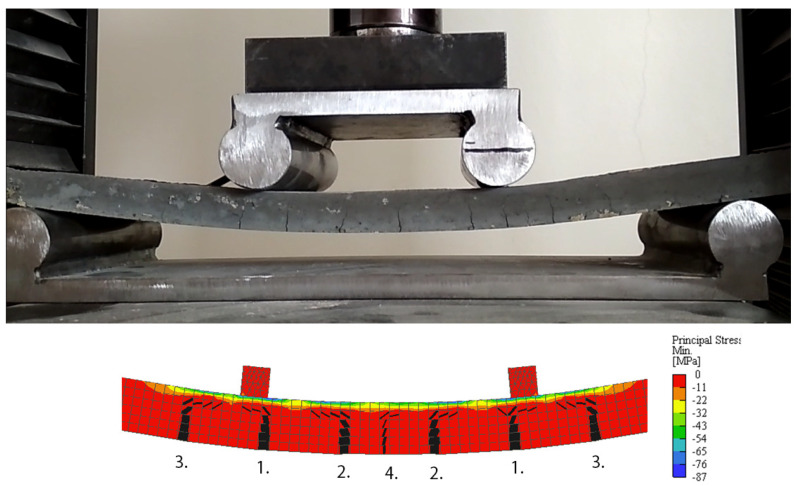
Comparison of crack development during real performed four-point bending test and detailed crack development output from the numerical analysis using Atena Engineering software. The order of the crack initiation is marked and the orientation and width of each crack is indicated. Distance of support is 300 mm, distance of loading support is 100 mm.

The four-point bending test was performed using LabTest 4.100SP1 testing machine on HPC slabs with dimensions of 360 × 100 × 18 mm reinforced with impregnated carbon rovings both with the smooth and modified surfaces. Five specimens with smooth surfaces and five (one specimen was discarded due to the inadequate position of the reinforcement) with modified surfaces were tested. The position of the reinforcement was exactly measured on cut specimens after the testing to account for slight inaccuracies. Figure 2 shows the crack development in the concrete matrix compared to one of the numerical models. The crack distribution of the numerical analysis is more symmetrical, but the representation is perfectly accurate. The most important thing was that the bonding conditions between the concrete matrix and textile reinforcement allowed the development of multiple cracks instead of a single crack opening.

**Figure 3:**
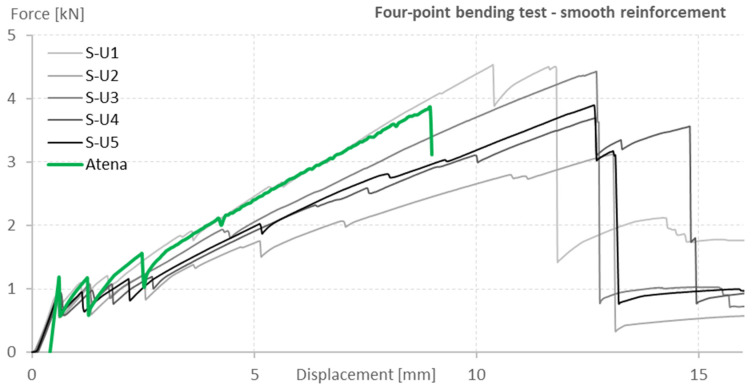
Load-displacement diagram comparison of the four-point bending test of specimens with smooth reinforcement and the numerical model prediction.

**Figure 4:**
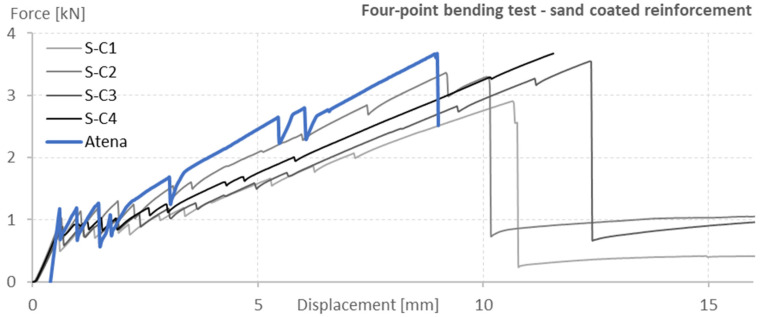
Load-displacement diagram comparison of the real performed four-point bending test of specimens with sand-coated reinforcement and the numerical model prediction.

The data acquired by the four-point bending test and the Atena Engineering numerical model were compared using load-displacement (L-D) diagrams. All fifteen numerical models were compared with the acquired data to evaluate which one of the models provided the best representation of the experiments. Figure 3 and Figure 4 present a comparison of the four-point bending test results and the prediction of the numerical model evaluated as the most relevant and selected for later use.

The numerical model shows higher stiffness compared to the tested specimens before the development of the first crack. The model cannot account for imperfections in the concrete surface, which can affect the positions of the loading supports and lead to load eccentricity. The curves from the numerical model were shifted so that the first cracks were formed at the same deformation as in the case of the tested specimens so that the development of the cracks and their opening could be compared. The differences between the four-point bending test and its numerical model were better described. The predicted failure at a lower displacement is due to the lower accuracy of the numerical model at a larger crack opening angle, which is more pronounced in thin structures.

## 3. Rigid Frame Corner Model

### 3.1. Shape Design

**Figure 5:**
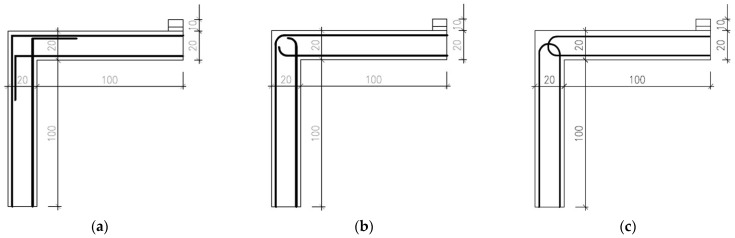
Three examples of reinforcement shape design. Schemes with dimensions for creating a numerical model in the Atena Engineering software. Sharply bent reinforcement (**a**); bent reinforcement (**b**); closed reinforcement (**c**).

The calibrated numerical model with adapted geometry was used to evaluate different types of reinforcement design [21]. All of them were subjected to loading in two directions, opening (negative bending moment) and closing (positive bending moment) of the rigid frame. The three most eligible variants are presented in Figure 5, all of which showed good load-bearing capacity. The most applicable shape of reinforcement was chosen based on its load-bearing capacity combined with the feasibility of manufacturing. The chosen shape was a variant shown in Figure 5c, which allowed for production in two separate identical parts.

### 3.2. Numerical Model

**Figure 6:**
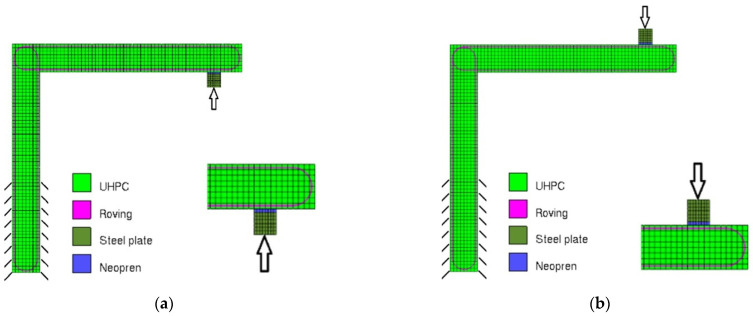
The numerical model of the rigid frame in Atena Engineering software with detailed view of the area of loading support: rigid frame opening (**a**); rigid frame closing (**b**). The arrows mark the position of the loading force.

An advanced model modified to match the experimental setup was prepared according to all parameters from the calibration described in the previous chapter. The rigid frame arms were the same length at 290 mm, the width of the element was 100 mm, and the thickness was 40 mm. The finite element network was created of 5 mm quadratic hexahedron elements. The lower end of the element’s vertical arm was fixed. The structure was loaded with a constant displacement applied to a midpoint of the support plate close to the free end of the sample. A neoprene layer between the steel support plate and the TRC sample was placed due to crack development under the support plate [24]. Figure 6a presents a model of rigid frame closing while Figure 6b—of its opening.

### 3.3. Results

**Figure 7:**
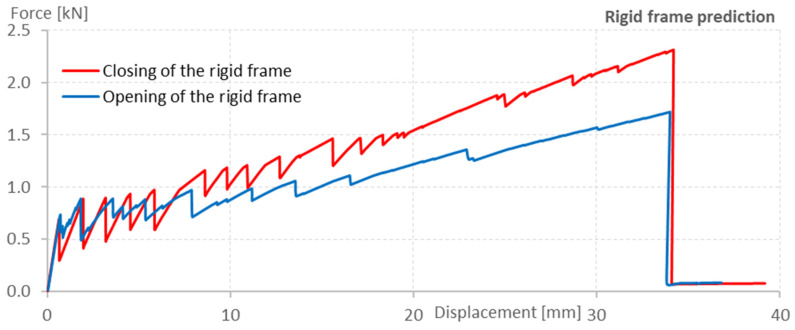
Numerical analysis prediction of the closing and opening of the rigid frames.

The numerical model was created to assess the mode of failure, theoretical crack development and opening, ultimate load-bearing capacity, and the maximum displacement in the moment of failure. A predicted load-displacement diagram is shown in Figure 7. The ultimate load-bearing capacity of the rigid frame while closing was estimated as 2.3 kN and opening as 1.7 kN, while the maximum displacement was approximately 34 mm in both cases. Failure of the rigid frame was both times predicted to be caused by the rupture of the textile reinforcement.

**Figure 8:**
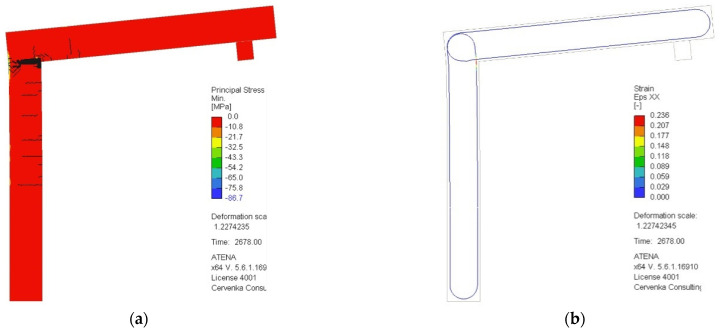
Mode of failure as predicted by the numerical analysis. Principal stress of the concrete matrix (**a**) and equivalent principal strain of carbon composite reinforcement (**b**) at the time of the failure.

Figure 8 presents the mode of failure of the opened rigid frame, as declared by the numerical analysis. The principal stress and the equivalent principal strain of the concrete matrix did not exceed critical values, while the ultimate tensile strength of the carbon roving was exceeded. In the calculation step after the peak load, a higher elongation of the reinforcement in one area is visible as a deformation higher than 0.015. That indicates bending failure caused by cracking of the reinforcement.

## 4. Rigid Frame Experimental Verification

### 4.1. Specimens Preparation

**Figure 9:**
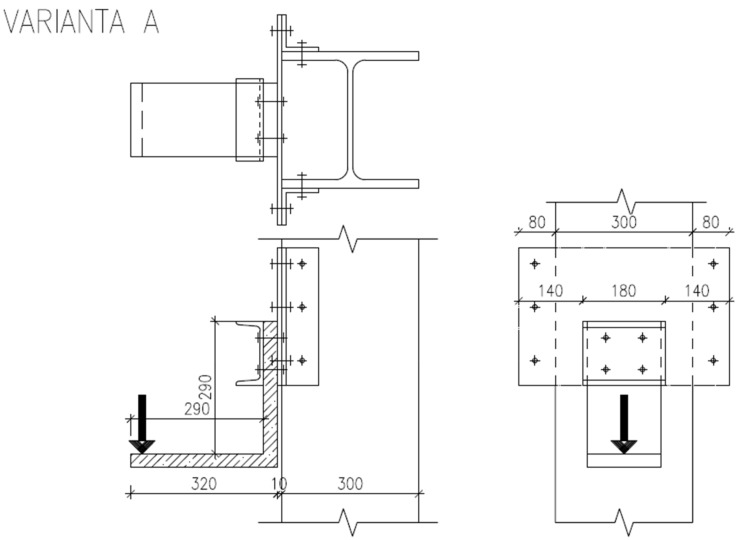
Design of the load-bearing destructive test for verification of the numerical model. The arrows mark the position of the loading force.

Two sets of specimens were prepared for the experimental verification of the behavior predicted by numerical analysis. Three specimens were prepared for the closing of the rigid frame and three for its opening. The rigid frames were subjected to load by a hydraulic press. The experimental setup is presented in Figure 9. The frame was attached to a steel structure, which allowed free movement of the rigid frame corner during either closing or opening of the frame. The experiment was performed to assess load-bearing capacity and compare it with the numerical analysis.

**Figure 10:**
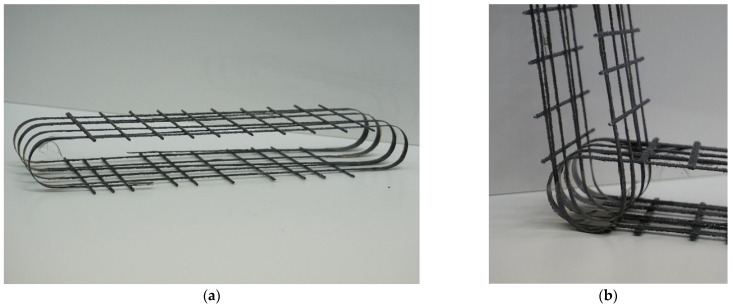
Rebar made of carbon roving (**a**) and detail presenting connection of rebars to form the reinforcement for the rigid frame (**b**).

The rigid frames were reinforced with the manufactured 3D textiles presented in Figure 10a. The shape of the rebars is based on the conclusions of the numerical analysis. This design also allows for manufacturing in parts, which are then connected at the required angle, as shown in Figure 10b. The rebars were manufactured in a laboratory. Carbon rovings were wound around a collapsible steel frame and laminated with epoxy resin. The rebars were then sand-coated as the resin began to harden to provide better bonding conditions between the rebar and the concrete matrix. After curing, the rebars were cut into 100 mm parts, so each rebar consisted of four rovings in a load-bearing direction connected by several rovings in the transverse direction.

**Figure 11:**
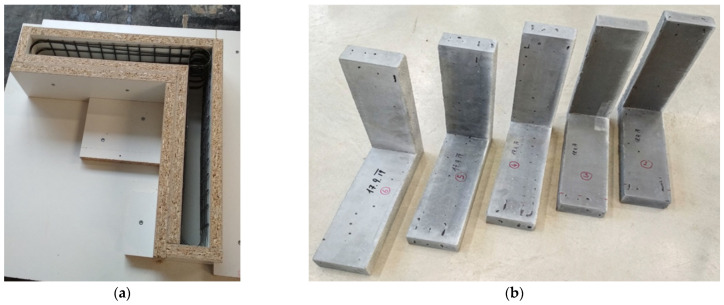
Formwork with installed reinforcement (**a**) and finished specimens (**b**).

Individual rebars were interlocked into the designed shape and placed inside formwork made of laminated boards. The position of reinforcement was secured on its own due to the small distance between the reinforcement and the form walls. Transverse rovings, which were not tensioned during the opening and closing of the rigid frame, were placed over the longitudinal rovings and were in direct contact with the form wall. This configuration provided a constant concrete cover of approximately 2 mm of the longitudinal rovings, which were tensioned during the opening and closing of the rigid frame. No additional spacers were necessary for reinforcement positioning. Compacting the concrete with vibrations would cause the rebars to float, so self-compacting concrete was used. All specimens were 100 mm wide with a thickness of 40 mm. Prepared formwork and finished specimens are shown in Figure 11.

### 4.2. Loading Proposal

The experimental loading of the rigid frame was designed according to the Czech technical standard ČSN 73 2030. The standard prescribes a method to calculate levels of force to which the tested element is subjected. Since the rigid frame specimens were loaded by the constant increment of displacement, the levels of force were recalculated to levels of displacement based on the numerical analysis. However, the designed displacement at the beginning of the experiment did not yield the required loading force due to inaccuracies such as subsidence of the press and deformation of the distribution plate. The loading diagram was, therefore, recalculated to match the designed levels of force. Loading was done using Inova 50kN hydraulic linear actuator.

### 4.3. Monitoring Proposal

To monitor the experiment, two potentiometers were placed under the specimen on both sides under the loading support to detect uneven deformation of the specimen. One other potentiometer was placed on the other side of the specimen to monitor that the specimen was not slipping from its fixed position. The monitoring was complemented by the DIC (digital image correlation). The DIC is based on the sequential scanning of the examined object by a digital camera and the calculation of displacement vectors. Other mechanical magnitudes can then be calculated in post-processing. To achieve better results, contrast colors were applied to specimens for better recognition in post-processing. The DIC enabled a more comprehensive analysis of the course of loading at any point. The main reason to employ the DIC was to monitor crack openings and the rotation of the specimens.

## 5. Results and Discussions

**Figure 12:**
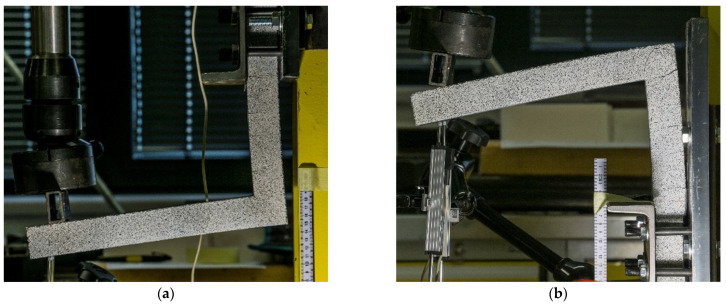
Opening of the rigid frame (**a**) and closing of the rigid frame (**b**).

Six specimens (two sets of three) were tested during the experiment until their collapse. Figure 12 shows the advanced state of both the closing and the opening of the rigid frames right before their failure. The most important monitored results were the crack development and opening, mode of failure, ultimate load-bearing capacity, and displacement. Figure 12 also shows a rotation of the loading support resulting in changing distance at which the force was applied. This issue was resolved with the data obtained with the DIC.

The experimental closing and opening of the L-shaped rigid frame was performed according to the technical standard ČSN 73 2030, which sets the limit of displacement as 1/50 of the span. The data acquired after the displacement exceeded this limit was used to examine the ductility of the structure and to evaluate the accuracy of the numerical analysis. The load-bearing capacity of the rigid frame was calculated based on the values of the loading force when each of the specimens reached the limit of displacement. The loading force applied to the specimens was converted to the bending moment acting on the corner and the vertical part of the specimens. Table 3 and Table 4 present the measured values, variation coefficients, and calculated characteristic and design value of the load-bearing capacity.

**Figure 13:**
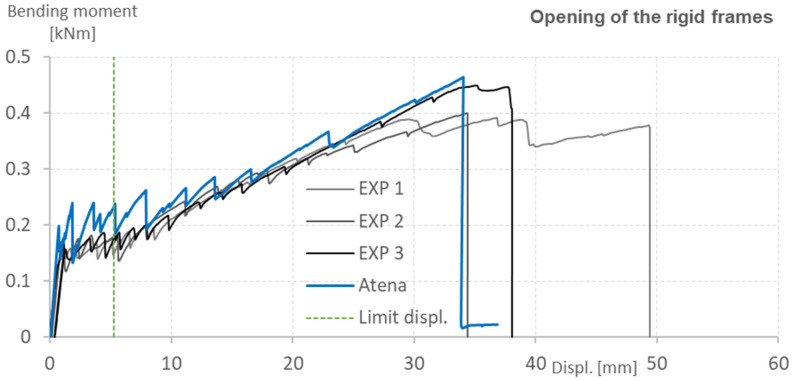
Opening of the rigid frames. Comparison of numerical analysis prediction and experimentally tested specimens.

**Figure 14:**
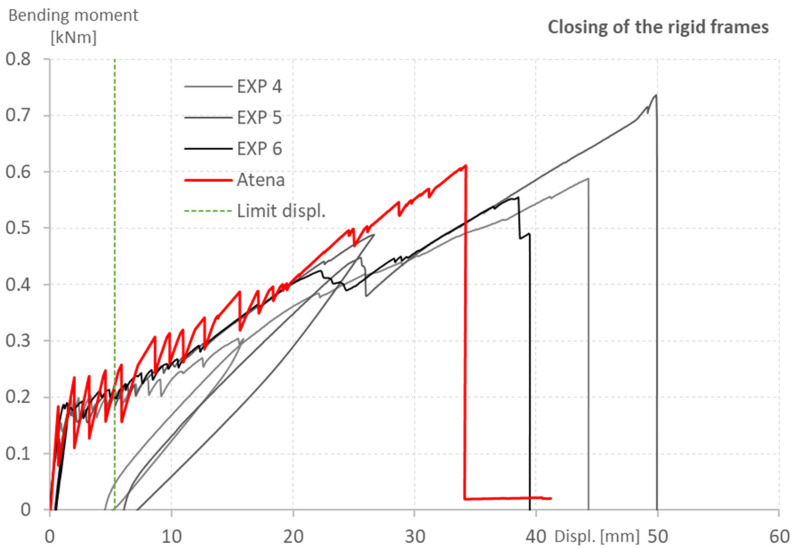
Closing of the rigid frames. Comparison of numerical analysis prediction and experimentally tested specimens.

The results of the experiment are shown in Figure 13 and Figure 14 in the form of bending moment-displacement diagrams. Because of the slight shifting of the loading support during the experiment, the exact position of the applied force was adjusted according to the DIC and was reflected in the calculations of the bending moment. The blue and red lines represent the predicted behavior calculated by the ATENA software, while the black and grey lines represent the tested specimens. The green dotted line shows the maximum displacement according to EN 73 2030, which specifies the limit as 1/50 of the span. The predicted results corresponded well with the experimental data, with only a small deviation at the first crack initiation.

The presented graphs show that the numerical analysis made in the ATENA software matches the experimental data very well, despite some obvious inaccuracies. The ultimate bending capacity of the tested specimens varied, especially in the case of the rigid frame closing. This was, however, expected due to the nature of the manually produced textile reinforcement, which rupture-marked the collapse of the element. The similarities were observed in the case of the displacement, which due to the same stiffness of all specimens, varied correspondingly.

The stiffness of the concrete matrix before the development of the first crack and involvement of the textile reinforcement (the first steep part of the graph up to almost 0.2 kNm) was predicted correctly. The development of the first crack, however, varied from the prediction and even between the specimens. This was also expected as issues, such as bubbles, blisters, or other defects which weaken the cross-section of the specimens, are common in the case of concrete elements.

The subsequent stiffness of the element after the initiation of crack development (gradually rising curve with several peaks marking each crack development) was also predicted correctly. Most importantly, the frequency of crack development was predicted with great accuracy, despite the fact that the prediction showed a more significant decrease in force after the development of each crack. This indicates that the bonding conditions could still be slightly improved to register the involvement of the textile reinforcement sooner after the crack initiation.

Two specimens were unloaded during testing of the rigid frame closing to show the plastic deformation of the specimens. The development of cracks led to local plastic deformation of the carbon textile reinforcement impregnated with epoxy resin. The unloading of the specimens had no impact on the ultimate strength or the mode of failure of the tested specimens.

**Figure 15:**
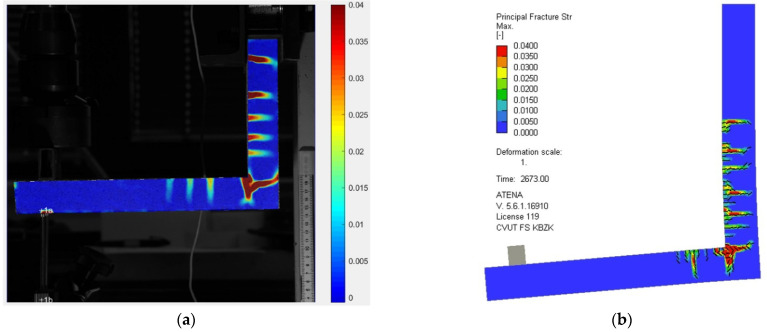
Comparison of the crack development and opening during the opening of the rigid frame. Principal strain of the experimental setup as captured by the DIC (**a**) and image depicting numerical strain predicted by the numerical analysis (**b**).

**Figure 16:**
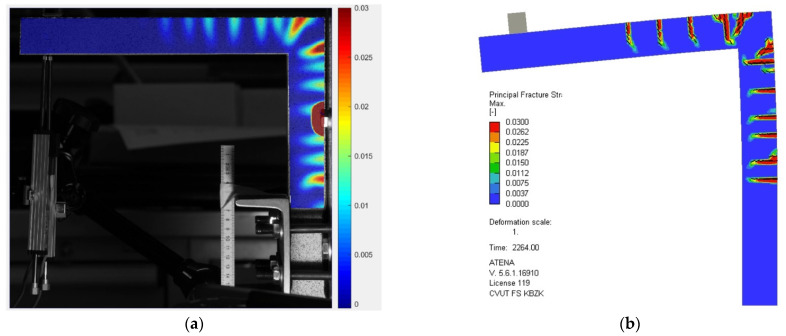
Comparison of the crack development and opening during the closing of the rigid frame. Principal strain of the experimental setup as captured by the DIC (**a**) and image depicting numerical strain predicted by the numerical analysis (**b**).

Figure 15 and Figure 16 present the comparison of crack opening as recorded by the DIC and as predicted by the numerical analysis. The prediction exactly matches the real crack development and opening, especially in the vertical and the corner part of the rigid frame, for both the closing and opening of the rigid frame. The numerical model predicted fewer cracks on the horizontal arm, but the prediction is overall perfectly accurate.

## 6. Conclusions

In this study, experimental verification of the rigid frame made of high-performance concrete reinforced with carbon textile reinforcement and numerical model calibration of this experiment was examined and discussed. The textile reinforcement provides high tensile strength and great durability even with a minimal protective layer of the concrete matrix. However, the general understanding of the bonding conditions between the textile reinforcement and the concrete matrix required for the efficient and safe design of the load-bearing TRC structures is not yet complete.

The designed rebars made of carbon rovings impregnated with epoxy resin and coated with silica sand proved to be easy to manufacture and deploy, provided sufficient load-bearing capacity, and the development of multiple cracks in the concrete matrix indicated good cohesion between the reinforcement and the concrete matrix. The carbon textile reinforcement, despite its brittle failure, provides a significant load-bearing reserve between the serviceability limit state and the ultimate limit state. The TRC elements should, therefore, provide significant warning of their impending failure and should be considered for application in load-bearing structures. The numerical model made in the ATENA software calibrated with data acquired in material experiments and the four-point bending test of TRC slabs was able to predict the behavior of complex TRC structure with a high level of accuracy, including correctly predicted mode of failure and development of multiple cracks and their opening.

This study proves that the behavior of TRC structures with textile reinforcement very close to the element surface can be accurately predicted, including exact crack development and the mode of failure. The numerical model and especially its bonding conditions between the concrete matrix and the textile reinforcement were properly calibrated. This could significantly improve the design process of complex TRC structures. Additionally, this study presents a novel approach to the reinforcement of the rigid frame made of high-performance concrete. The individual 3D rebars made of carbon roving penetrated with epoxy resin and coated with fine-grained silica sand can be interlocked under almost any designed angle, which provides high variability in possible applications. Future work will focus on improving the textile reinforcement to have a more ductile behavior, as a brittle failure of the reinforcement is the most significant issue of the current design.

## Figures and Tables

**Table 1:** High-performance concrete mix composition.

Component	[kg/m^3^]
Cement I 42.5 R	680
Technical silica sand	960
Silica flour	325
Silica fume	175
Superplasticizers	29
Water	171

**Table 2:** Comparison of declared and measured values.

	Declared Value	Surface Treatment	Measured Value
Tensile strength	4400 MPa	Smooth	3506 ± 233 MPa
		Silica sand	3423 ± 241 MPa
E modulus	240 GPa	Smooth	247 ± 12 GPa
		Silica sand	267 ± 16 GPa

**Table 3:** Characteristic and design values for opening of the rigid frame.

Specimen Number	Limit Displacement M [Nm]	Average Value M [Nm]	Characteristic Value M_Rk_ [Nm]	Design Value M_Rd_ [Nm]
1	178	176 ± 3	164	109
2	177			
3	172			

**Table 4:** Characteristic and design values for closing of the rigid frame.

Specimen Number	Limit Displacement M [Nm]	Average Value M [Nm]	Characteristic Value M_Rk_ [Nm]	Design Value M_Rd_ [Nm]
1	196	204 ± 6	179	120
2	205			
3	211			

## Data Availability

The data presented in this study are available on request from the corresponding author. The data are not publicly available.

## References

[1] Hegger J., Zell M., Horstmann M. (2008). Textile reinforced concrete—Realization in applications. International Fib Symposium Tailor Made Concrete Structures: New Solutions for Our Society.

[2] Butler M., Mechtcherine V., Hempel S. (2010). Durability of textile reinforced concrete made with AR glass fibre: Effect of the matrix composition. Mater. Struct..

[3] Hempel R., Butler M., Hempel S., Schorn H. (2007). Durability of textile reinforced concrete. Spec. Publ..

[4] Peled A., Mobasher B., Bentur A. (2017). Textile Reinforced Concrete.

[5] Hegger J., Curbach M., Stark A., Wilhelm S., Farwig K. (2018). Innovative design concepts: Application of textile reinforced concrete to shell structures. Struct. Concr..

[6] Valeri P., Guaita P., Baur R., Ruiz M.F., Fernández-Ordóñez D., Muttoni A. (2020). Textile reinforced concrete for sustainable structures: Future perspectives and application to a prototype pavilion. Struct. Concr..

[7] Zeng J.-J., Zeng W.-B., Ye Y.-Y., Liao J., Zhuge Y., Fan T.-H. (2022). Flexural behavior of FRP grid reinforced ultra-high-performance concrete composite plates with different types of fibers. Eng. Struct..

[8] Cauberg N., Tysmans T., Adriaenssens S., Wastiels J., Mollaert M., Belkassem B. (2012). Shell Elements of Textile Reinforced Concrete Using Fabric Formwork: A Case Study. Adv. Struct. Eng..

[9] Simonsson E. (2017). Complex Shapes with Textile Reinforced Concrete. Master’s Thesis.

[10] Pohoryles D.A., Bournas D.A. (2020). Seismic retrofit of infilled RC frames with textile reinforced mortars: State-of-the-art review and analytical modelling. Compos. Part B Eng..

[11] Filippou C., Furtado A., De Risi M.T., Kyriakides N., Chrysostomou C.Z. (2022). Behaviour of Masonry-Infilled RC Frames Strengthened Using Textile Reinforced Mortar: An Experimental and Numerical Studies Overview. J. Earthq. Eng..

[12] Yi W.-J., He Q.-F., Xiao Y., Kunnath S.K. (2008). Experimental study on progressive collapse-resistant behavior of reinforced concrete frame structures. ACI Struct. J..

[13] Feng D., Kolay C., Ricles J.M., Li J. (2016). Collapse simulation of reinforced concrete frame structures. Struct. Des. Tall Spec. Build..

[14] Nilsson I.H., Losberg A. (1976). Reinforced concrete corners and joints subjected to bending moment. J. Struct. Div..

[15] Šmejkal J., Procházka J. (2009). Navrhování s použitím modelů náhradní příhradoviny. Beton TKS.

[16] Semrád K., Szücs C. (2009). Řešené příklady betonových konstrukcí pomocí příhradové analogie. Fak. Staveb. ČVUT V Praze.

[17] Vlach T., Řepka J., Hájek J., Fürst R., Jirkalová Z., Hájek P. (2020). Cohesion Test of a Single Impregnated Ar-Glass Roving in High-Performance Concrete. Staveb. Obz. Civ. Eng. J..

[18] Hajek P., Novotná M., Chira A., Fiala C., Vlach T., Laiblová L. Challenge of textile reinforced high performance concrete for sustainable construction. Proceedings of the Concrete-Innovation and Design, Fib Symposium.

[19] Chira A., Kumar A., Vlach T., Laiblová L., Hajek P. (2016). Textile-reinforced concrete facade panels with rigid foam core prisms. J. Sandw. Struct. Mater..

[20] Řepka J., Vlach T., Laiblová L., Hájek P., Ženíšek M., Kokeš P. (2017). Thin Lightweight Panels Made of Textile Reinforced Concrete. Solid State Phenom..

[21] Žalský J., Vlach T., Laiblová L., Jirkalová Z., Řepka J., Hájek P. (2019). Numerical Analysis of Rigid Frame Joint with Textile Carbon Reinforcement. Solid State Phenom..

[22] Cervenka V., Cervenka J., Pukl R. (2002). ATENA—A tool for engineering analysis of fracture in concrete. Sadhana.

[23] Červenka V., Jendele L., Červenka J. (2000). ATENA Program Documentation—Part 1.

[24] Vlach T., Laiblová L., Ženíšek M., Řepka J., Hájek P. (2018). Soft Insert for Support Modeling of Slightly Textile Reinforced Concrete. Key Eng. Mater..

